# Radiographic alveolar bone level and levels of serum 25-OH-Vitamin D_3_ in ethnic Norwegian and Tamil periodontitis patients and their periodontally healthy controls

**DOI:** 10.1186/s12903-019-0769-6

**Published:** 2019-05-14

**Authors:** Vimalan Ketharanathan, Gerald R. Torgersen, Beáta Éva Petrovski, Hans R. Preus

**Affiliations:** Department of Periodontology, Institute of Clinical Odontology, Faculty of Dentistry, University of Oslo, POB 1109 Blindern, 0317 Oslo, Norway

**Keywords:** Periodontal disease, Vitamin D, Bone loss

## Abstract

**Background:**

Studies suggest association between low serum 25-OH-Vitamin D_3_ (VitD) and chronic destructive periodontal diseases. The main sources of VitD is sun exposure and fat fish. Subjects with dark skin will therefore generate less VitD as response to sun exposure. The aim of the study was to assess the radiographic bone level and levels of serum VitD in ethnic Norwegian and Tamil periodontitis patients and their respective healthy controls.

**Methods:**

Twenty-seven Tamil periodontitis patients living in Norway were compared to 21 Tamil controls as well as to 21 Norwegian periodontitis patients and 23 Norwegian controls. Marginal bone level was diagnosed on radiographs. VitD levels were diagnosed in blood samples by high-performance liquid chromatography-mass spectrometry.

**Results:**

VitD levels were lower in Norwegian periodontitis patients than in controls, while no significant differences were observed between Tamil periodontitis patients and controls despite the significant difference between RBL between the periodontitis patients and controls in both groups. When calculating the odds ratio for having periodontal disease in both populations together, it appeared that one unit increased serum VitD (i.e. 1 nmol/L) decreased the odds of having radiographic bone loss by 4%.

**Conclusion:**

According to logistic regression, and after correcting for confounding factors, VitD levels showed significant association with the presence of periodontitis, as expressed by radiographic bone loss, in all patients combined.

**Electronic supplementary material:**

The online version of this article (10.1186/s12903-019-0769-6) contains supplementary material, which is available to authorized users.

## Background

Studies on the pathogenesis of periodontal diseases have mostly focused on bacterial etiology based on several different infection hypotheses [1]. Consequently, intervention studies [2, 3] have mainly focused on oral hygiene, mechanical debridement and antibiotic strategies [4–6] to reduce sub- and supragingival bacterial load and composition.

However, mechanisms for reduced ability to regenerate bone after destruction [7, 8] may also contribute to the pathogenesis of periodontitis. One of the factors of recent interest has been serum levels of 25-OH-Vitamin D_3_ (VitD).

VitD, and its locally active metabolite 1,25-dihydroxyvitamin D_3,_ have been reported to interfere with insulin production [9] and type 2 diabetes [10], have anti-inflammatory function [11] as well as ability to suppress secretion of Receptor activator of nuclear factor κ-B ligand, Tumor necrosis factor α, Interleukin 6 and 17 and Interferon-γ in rheumatoid arthritis patients [12]. Following this wide range of effects on infection mediators, VitD has also been reported directly or indirectly associated with cardiovascular disease [13], rheumatoid arthritis [14, 15], osteoporosis [16, 17] as well gingival inflammation [18] and destructive periodontal disease [19–24].

Sri Lankan Tamils have been reported especially prone to periodontitis [17, 25]. A large group of political refugees from Sri Lanka (Tamils) has been living in Norway for many years and their dark skin causes a reduced ability to produce sun-generated VitD when relocated to less sun-rich geographical locations like Norway [26]. The aim of the present study was therefore to assess the radiographic bone level (RBL), as the surrogate for periodontitis, and levels of serum VitD in ethnic Norwegians and Norwegian relocated Tamil periodontitis patients, and their respective healthy controls.

## Methods

### Sample size and power calculation

Two groups of Sri Lankan Tamil refugees, living in Norway, were compared on a possible association between RBL and blood levels of VitD. Focus was variable “VitD measurement” (D). Based on results from a study of Sri Lankans in Oslo, Norway and Sri Lanka [26], it was assumed that the standard deviation (sd.) of D would be 22.8 in both groups.

N independent samples t-test, with 5% significance level, was used when comparing mean D in the two groups. The study should hold at least 80% test-power to detect a difference in mean D between groups, if the true mean difference in D was at least 20. It was then shown that at least 21 males had to be included in each group. The assumption that the difference in mean X between the groups was realistic; it was decided to include at least 21 males in each group. Since the sd. of D in a Norwegian population is 24.3 [26], the same calculation and assumptions were done for the Norwegian control populations arriving at the same number of required patients and controls.

### Population

Based on the above calculation, 21 ethnic Norwegians and 27 ethnic Tamil political refugees living in Norway for more than 5 years, all with the clinical diagnosis “established periodontal disease” [27], were recruited as test populations for this case control study. Twenty-three Norwegians and 21 ethnic Tamil political refugees living in Norway, all periodontally healthy, were recruited as control subjects. “Periodontally healthy” was defined as no loss of attachment (no previous history of periodontal disease), no pocket depths > 3 mm. The periodontal patients were recruited from two private practices in the greater Oslo area, whereas the Tamil and Norwegian control subjects were recruited from the register of patients and staff at the Institute of Clinical Odontology, Dental Faculty, University of Oslo, Norway. The Regional Committee for Medical & Health Research Ethics, Oslo, Norway (REK) approved the study (Approval No. 2015/443). The study was performed during autumn 2015.

The inclusion criteria for both populations were males, 30–70 years, diagnosis of “established periodontitis” based on clinical attachment level (CAL) and probing pocket depth (PPD) measurements, i.e. “the presence of CAL ≥ 6 mm in 2 or more teeth and one or more sites with PPD ≥ 5 mm” [27]. However, it must be remembered that these clinical measurements only reflect the history of periodontal destruction and not an ongoing periodontitis. These participants will further be referred to as the “test groups”. Both light smokers and non-smokers were recruited. Patients were asked if they smoked, and if so, how many cigarettes they consumed per day. None of the included test or control persons used smokeless tobacco. Participants had to be willing to sign an informed consent form. Exclusion criteria were any general disease or condition, drug or alcohol abuse.

#### Clinical screening of periodontal disease

One periodontist (HRP) and one periodontal specialist candidate (VK) screened and included all periodontitis patients and controls based on the criteria mentioned above [27]. Prior to screening, VK was calibrated to HRP on both reading PPD and CAL. The procedure is described in Preus et al. 2013 [28]. Since the focus of this study was on VitD’s bone affecting properties and periodontal destruction, radiographic identification of marginal bone level (RBL) was employed as the surrogate for the periodontal diagnosis.

#### Radiograpic examination

Radiographic images were obtained by the Soredex Digora Optime (Soredex, Finland) digital phosphor storage plate system and Planmeca intra (Planmeca Oy, Finland) X-ray units. For full radiographic examination, 10 phosphor storage plates were used in Eggen’s holders [29]. For BiteWing radiographs (BW), Quick Bite® holders (Hawe Neos, Kerr, Switzerland) were used, and all readable sites from the distal surface of canines to the mesial surface of second molars were registered. Radiographic images were calibrated and exported by the Digora® for Windows software to 8bit grey scale Tagged Image File Format files. The calibration information in the image files (i.e. Exif information) were used to obtain the coordinates of the sites. The latest version of the *ImageJ* image processing and analysis software program [30] was used for the experiment. An application (plugin) to this program had been developed [31] with the intent to semi-automatically register the ratio between the bone level and the corresponding length of tooth in longitudinal studies. The plugin allowed for just pointing and clicking on the Cemento-Enamel Junction (CEJ), alternatively the Restoration Margin (RM) and Alveolar Crest (AC) to digitally register these points. This technique has proven more accurate, versatile and easy to use [31] than the original *ImageJ* technique [30] even for direct measurements like in the present study.

In short, the computer application automatically stores the marked coordinates (relative to the upper left corner of the image) and projects them onto an imaginary vertical axis parallel to the vertical dimension of the receptor [31]. The coordinates, unit of length, time and date of measurement were automatically stored in a text file, which was imported into an EXCEL spreadsheet to automatically calculate the distance from the RBL.

Only one researcher (VK), who had been trained extensively in the above-described reading method, examined the radiographs. After training, VK was presented two sets of masked full mouth radiographs (10 radiographs each) in addition to 10 BW radiographs (2 radiographs in each) from randomly chosen patients from the Clinic of Periodontology. These were read once before being re-masked and re-examined. Thus, altogether 40 radiographs were examined twice by the above described technique, and the intra class correlation [32] was calculated to be 0.91.

The study radiographs from patients and controls were blinded and randomized using a macro (program code) written in the *ImageJ* [30] macro language.

The image files were numbered, randomly reorganized and blinded to the reader. Bone level was measured as the distance from the CEJ or RM to the most coronal level of the AC. The point where the periodontal space retained its normal radiographic width was considered the alveolar crest, and infrabony pockets were measured to the point of the most apically advanced radiolucency [29]. All sites on the x-rays not clearly identifiable, as well as any other uncertainty regarding identification of the measured sites were excluded from the study as non-readable sites.

Following the reading of images, a second macro was used to replace the coded filename with the original patient reference in the result file using the key file. Then the result file was sorted by patient and tooth number for further analysis by the IT engineer (GRT).

#### VitD estimation in blood

Dried Blood Spots analyses of blood levels of VitD were employed in this study.^1^Finger-prick blood samples on filter paper cards^2^ were obtained chairside by VK, providing two drops of blood (100 μL) per patient/control. The filter papers with blood were stored in the laboratory at − 80 °C until analysis of VitD could be performed on all samples. Samples were analyzed by one batch high-pressure liquid chromatography-atmospheric pressure chemical ionization-mass spectrometry, which has been regarded as the most valid method for VitD analyses [33]. The method of analysis has a precision and accuracy of 8% and of 98.7% respectively where the latter is assessed in an external quality control system, “Vitamin D External Quality Assessment Scheme” [34]. These VitD measurements reflect the actual level of VitD in the patients’ serum in contrast to the RBL measurements, which reflect the history of periodontal destruction and not an ongoing periodontitis.

#### Statistical analyses

Descriptive statistical analysis was performed. Mean + SD and median/interquartile ranges (IQR) are presented. Normality of continuous variables was tested on histogram, Q-Q plot and by Shapiro-Wilk and Kolmogorov-Smirnov test. Since the normality assumption was not satisfied, Mann-Whitney U test was used to compare differences in the medians of the two groups. Chi-square (*χ*2) test was used to test the differences of the distribution of categorical variables.

Simple logistic regression was used to study the relationship between VitD level and periodontal disease. Only crude odds ratio (OR) and its 95% confidence interval (CI) is presented. Age and ethnicity were not included in the regression model since they showed no association with the disease, neither in the crude nor in the adjusted analysis.

Significance limit was set as *P* < 0.05. All statistical analyses were performed using the Statistical Package for STATA (Stata version 14.0; College Station, TX, USA).

## Results

Two and four of the Tamil and Norwegian periodontitis patients smoked respectively, and those who did reported a consumption of less than five cigarettes per day. Neither Tamil nor Norwegian control patients reported to be current smokers. The median age of the participants was 46 years (IQR: 39–50; range: 30–70). The age was not significantly different between the study groups (Additional file 1: Table S1).

**Fig. 1:**
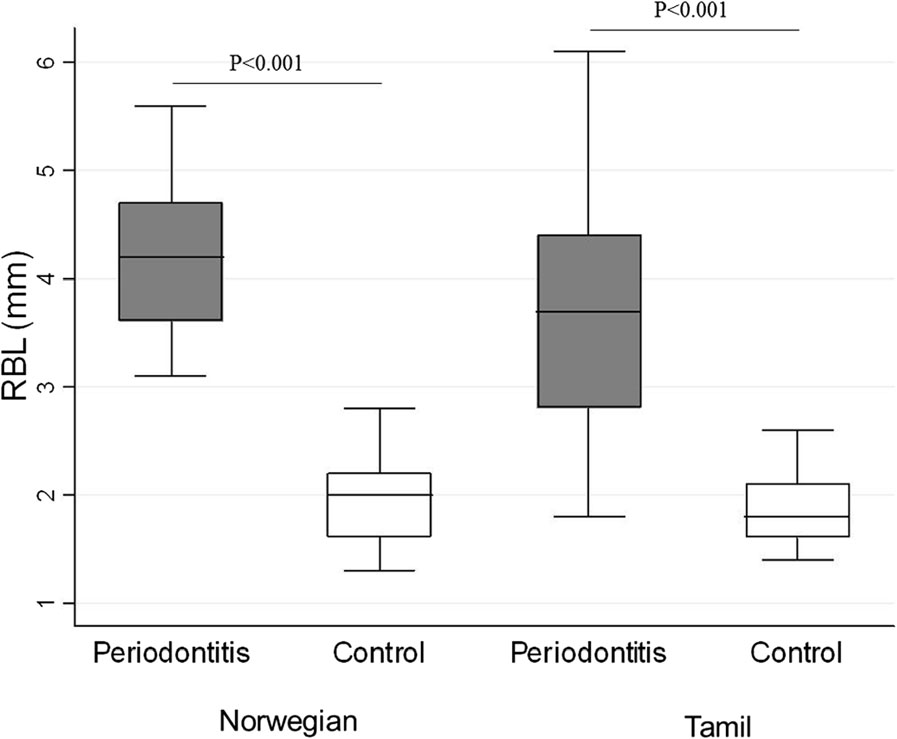
The difference between RBL (mm) in periodontitis patients and healthy controls in both ethnic groups

**Fig. 2:**
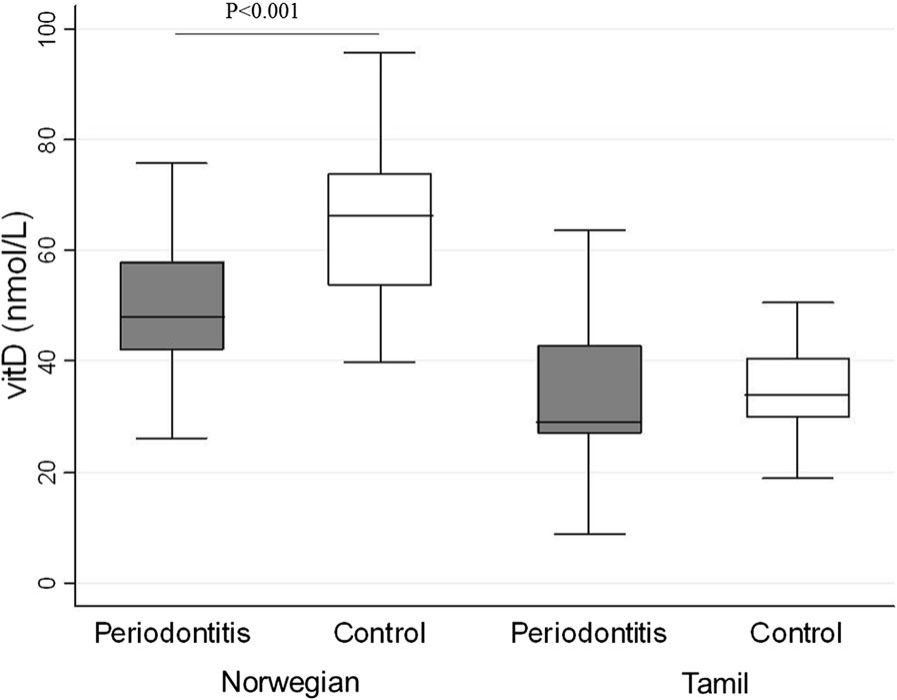
The relationship between vitamin D (VitD (nmol/L)) in periodontitis patients and healthy controls in both ethnic groups

The clinical selection of periodontitis patients and healthy controls was confirmed as RBL was significantly higher in both ethnic groups among those with periodontitis as compared to their healthy controls (*P* < 0.001) (Fig. 1. Additional file 1: Table S1). VitD levels were significantly lower in Norwegian periodontitis patients than in their healthy controls (*P* < 0.0001), while no significant differences were observed among the Tamils (Fig. 2 and Additional file 1: Table S1). VitD level showed a statistically significantly association with periodontal disease in both populations combined, as one unit (1 nmol/L) increase could decrease the odds of having periodontal disease by 4% (OR, 95% CI: 0.96(0.94–0.99) *p* < 0.01).

The raw data from this study, as well as the logistic regression are presented on the home page of HPreus: https://www.odont.uio.no/iko/english/people/aca/hpreus/index.html

## Discussion

In the present, cross sectional study, VitD levels were significantly lower in the Norwegian periodontitis group than in their healthy controls. There was a small difference in the Tamil periodontitis and control groups as well, although not statistically significant. In these populations combined, the odds ratio for having periodontal disease was significantly reduced with increasing levels of VitD.

RBL represents the history of destructive periodontitis in each patient, and does not indicate active periodontal disease, whereas VitD levels reflect an actual status being subject to seasonal fluctuations and dietary habits. Seasonal fluctuations in VitD levels, due to varying sun exposure seem more important than dietary habits, which are more stable ingredients in human lives. According to a study by the University of Oslo, Norway and the Norwegian Institute of Public Health, Sri Lankan men living in Norway increased their VitD status from an average of 30.5 nmol/L following winter (early April) to 38.9 nmol/L in summer (June), i.e. an increase of 8.4 nmol/L during spring [26]. This was comparable to the reduction recorded (i.e. 9.1 nmol/L) in ethnic Norwegians (men and women) in the period August/September to January [35]. Since there are no systematic registrations of seasonal variations in VitD in Sri Lankans living in Norway or ethnic Norwegians, the above reports were the best we could find. However, the above variations accounted for a little shy of 10 nmol/L between seasons in both populations. Although only based on these observations, this might indicate that a person of low VitD remains in the lower channel, and vice versa. A hypothesis might therefore be that people have an individual natural history of varying low or varying high VitD, possibly connected to accompanying diseases when low. When compensated for by VitD supplements, this might change this hypothetical receptivity of VitD associated traits [36].

For ethical reasons and since there was no need for extensive radiological examination in the control groups, only recently obtained BW radiographs were used for identifying bone level. Most Norwegians, including our control populations, attend their dentist once or twice a year, where also BWs are obtained, and therefore we were able to get recent BWs from all control persons. For therapy in periodontitis patients, the Norwegian health authorities require a full radiographic status (10 radiographs), since Norway offer considerable public refund for this kind of treatment. Proper documentation of disease is therefore mandatory. Thus, RBL levels of all periodontitis patients in this study were registered on these recently obtained radiographs, which were taken for therapy purposes. The question of full mouth - versus partial mouth recordings has been explored by Kingman et al. [37] suggesting that partial mouth examination suffices in identifying periodontal disease in patients.

Another major challenge affecting measurements of bone level changes on radiographs is the angle by which the radiographs have been obtained. A deviance in angle between the beam direction in BW and full tooth radiograph is obvious, especially in the upper canine region. This problem has been addressed by Preus et al. 2015 [31] and it was shown that a change in beam angle (α) of 30^o^ resulted in an 11% increase of RBL readings of an actual bone level of 7 mm. The increased reading will be reduced as the actual bone level is reduced, and at 2–3 mm an α of 30^o^ will not increase RBL significantly. Therefore, comparing the disease - with the control groups by radiographs as described should be acceptable.

It may be argued that the examiner would recognize the control subjects on the BW radiographs. However, BW included Tamil as well as Norwegian control subjects. Moreover, the radiographs were masked prior to - and de-masked following the reading by the IT engineer (GRT). Therefore, the examination should be regarded as masked.

In this study, smoking was of a minor consequence since only very few of the periodontitis patients smoked, and those who did, smoked < 5 cigarettes/day. It is not customary to smoke in Norway, and in 2018 less than 12% of Norwegians smoked on a daily basis [38], which explains the low rate of smokers in our populations. The reason inclusion allowed smokers was that it was expected difficult to find enough Tamil healthy controls based on the description of this population in “the natural history of periodontal disease” [17]. Never the less, none of the healthy controls, regardless of ethnicity, smoked. The two ethnic test groups could therefore be regarded as equal in this regard as well.

A study by the Norwegian Institute for Public Health [26] showed that Tamils living in Sri Lanka and Norway had an average VitD blood level of 54.2 and 31.5 nmol/L respectively. The Tamil subjects (periodontitis patients and controls alike) in the present study were diagnosed with an average VitD level of 33.9 nmol/L, which was in concert with the above mentioned study [26], as with another study of Indian Tamils as well [39]. In the same study [26], ethnic Norwegians were diagnosed with an average VitD level of 74.6 nmol/L whereas the present study reports an average VitD level of 57.7 nmol/L. This difference might be explained by the timing of blood sampling of the present study which was in the autumn of 2015, following a summer that had been less sunny and significantly colder and rainier than normal. One should then expect the Tamils to suffer from the same lack of sun. However, as the blood VitD decreases there are compensatory mechanisms preventing the fall of VitD to critically lower levels [40] which might explain the differences between the ethnic groups exposed to the same amount of sun.

In Norway VitD levels < 50 nmol/L; 50–75 nmol/L; 80–120 nmol/L are respectively regarded as deficiency, suboptimal and optimal. None of the study participants reported to have taken any medicinal VitD supplements for 3 months prior to this study, although it is customary for Norwegians to take cod liver oil for daily VitD and Ω_3_ supplements during the winter. Nutritional habits were not explored to the full, but an inclusion regime based on nutritional habits would make this study impossible since dietary habits between, as well as within, these populations would vary considerably.

Low levels of VitD have been associated with periodontal disease [19–24, 41, 42]. However, in these studies only soft tissue measurements (PPD and CAL) have been used as the basis for the diagnosis. As described by Preus et al., [4] soft tissue reactions like gingival inflammation and edema may variably contribute to inflate measurements of PPD and CAL. Therefore, to avoid contamination of various soft tissue reactions, like gingivitis associated with insufficient oral hygiene, only radiographic signs of bone loss were used as surrogate for the diagnosis of periodontitis in the present study. Again, it must be emphasized that even RBL only reflects the history of the sum of destructive events that has happened to the patient, and not the indication of an active disease.

## Conclusions

The findings in the present study seem at a first glance to be in agreement with studies showing that subjects with periodontal disease have lower levels of VitD than those with better periodontal conditions [19–24, 41, 42], at least in Caucasians. However, the findings also suggest a more complex, but less clear association between VitD and periodontal disease, expressed as surrogate RBL, than those previously published, as both Tamil periodontitis patients and healthy controls had comparable VitD status. Although only based on two different ethnic groups, and with all due caution, the results from the present study might suggest that VitD have different reference values and effect on periodontal health in different ethnic groups, and that the ethnicity being not associated with disease might be “masked” by the preponderance of the Norwegian difference in VitD levels.

## Additional file

Additional file 1: Differences in the radiographic bone level (RBL(mm)), vitamin D (VitD (nmol/L)) level and age of Norwegian and Tamil periodontitis patients and their respective healthy controls. (DOCX 18 kb)

## Abbreviations

AC: Alveolar crest
BÉP: Beáta Éva Petrovski
BW: BiteWing
CAL: Clinical Attachment Level
CEJ: Cemento Enamel Junction
GRT: Gerald Ruiner Torgersen
HRP: Hans Ragnar Preus
nmol/L: nanomol/Liter
PPD: Probing Pocket Depth
RBL: Radiographic Bone Level
REK: Regional Committee for Medical & Health Research Ethics
RM: Restoration Margin
Sd/SD: Standard deviation
SPSS: Statistical Package for the Social Sciences
VitD: Vitamin D (25-OH-D_3_)
VK: Vimalan Ketharanathan

## Footnotes

1 Vitas Ltd., Oslo, Norway. www.vitas.no.

2 903 Sample Collection Cards, GE Healthcare, Oslo, Norway
