# Supplementary material for: Radiographic alveolar bone level and levels of serum 25-OH-Vitamin D3 in ethnic Norwegian and Tamil periodontitis patients and their periodontally healthy controls
Source: BMC Oral Health. 2019 May 14;19:83. doi: 10.1186/s12903-019-0769-6 (PMC6518642; doi:10.1186/s12903-019-0769-6)
Supplement: Supplementary file 1 — Differences in the radiographic bone level (RBL(mm)), vitamin D (VitD (nmol/L)) level and age of Norwegian and Tamil periodontitis patients and their respective healthy controls. (DOCX 18 kb) [file 12903_2019_769_MOESM1_ESM.docx]

**Additional table 1. Difference in the radiographic bone level (RBL (mm)), vitamin D (VitD (nmol/L)) level and age of Norwegian and Tamil periodontitis patients and their respective healthy controls.**

|  | Norwegian  N=44 | | Tamil  N=48 | |
| --- | --- | --- | --- | --- |
|  | Periodontitis  N=21 | Controls  N=23 | Periodontitis  N=27 | Control  N=21 |
| Age (years)  Mean+SD  Median (IQR)  Range | 52.05+8.99  51.00 (46.00-61.00)  37.00-66.00 | 50.30 + 13.13  48.00 (38.00-64.00)  33.00-70.00 | 42.59 + 6.67  45.00 (39.00-48.00)  30.00-50.00 | 41.10 + 5.74  41.00 (37.00-47.00)  32.00-49.00 |
| RBL (mm)  Mean+SD  Median (IQR)  Range | 4.21 + 0.75  4.20 (3.60-4.70) ***p<0.001***  3.10-5.60 | 2.00 + 0.41  2.00 (1.60-2.20)  1.30-2.80 | 3.78 + 1.23  3.70 (2.80-4.40) ***p<0.001***  1.80-7.30 | 1.91 + 0.42  1.80 (1.60-2.10)  1.40-2.90 |
| VitD level (nmol/L)  Mean+SD  Median (IQR)  Range | 47.90 + 15.35  48.00 (42.00-58.00) ***p<0.001***  10.00-75.90 | 66.60 + 18.45  66.40 (53.70-74.10)  39.70-125.70 | 33.34 + 12.40  29.10 (26.90-42.90)  8.60-63.60 | 34.62 + 8.61  34.00 (29.90-40.60)  18.80-50.70 |
